# Sotagliflozin vs Dapagliflozin: A Systematic Review Comparing Cardiovascular Mortality

**DOI:** 10.7759/cureus.45525

**Published:** 2023-09-19

**Authors:** Nandhini Iyer, Sally Hussein, Sanjana Singareddy, Vijay Prabhu SN, Arturo P Jaramillo, Mohamed Yasir, Tuheen Sankar Nath

**Affiliations:** 1 Internal Medicine, California Institute of Behavioral Neurosciences & Psychology, Fairfield, USA; 2 Research, California Institute of Behavioral Neurosciences & Psychology, Fairfield, USA

**Keywords:** soloist-whf, mortality reduction, management of heart failure, heart failure with preserved ejection fraction, heart failure with reduced ejection fraction, hospitalization for heart failure, cardiovascular mortality, heart failure, dapagliflozin, sotagliflozin

## Abstract

After the debut of the results of the effect of Sotagliflozin on Cardiovascular Events in Patients with Type 2 Diabetes Post Worsening Heart Failure (SOLOIST-WHF) and Sotagliflozin in Patients With Chronic Kidney Disease and Type 2 Diabetes (SCORED) trials at the American Heart Association’s 2020 Scientific session, sotagliflozin became the first drug and the third sodium glucose co-transporter-2 (SGLT-2) inhibitor to be approved for heart failure (HF) across the spectrum of ejection fraction (EF). In light of this recent major U.S. Food and Drug Administration (FDA) approval of sotagliflozin, we conducted a systematic review to compare the cardiovascular mortality rates between sotagliflozin and dapagliflozin in patients with HF.

To find relevant articles, we extensively searched major research literature databases and search engines such as PubMed, MEDLINE, PubMed Central, Google Scholar, Embase, and Cochrane Library. We compared the results of significant trials involving sotagliflozin with the trials studying dapagliflozin to provide comprehensive mortality results of both drugs. The results showed that the timely initiation of sotagliflozin in HF cases significantly reduces cardiovascular mortality, hospitalizations, and urgent HF visits. Comparative trials with dapagliflozin indicate enhanced mortality reduction associated with greater initial symptom burden. The results of these major trials cannot be overlooked due to the large size of the combined trials, the randomized design, and the high standards with which they were conducted. The pathophysiology behind the cardioprotection offered by these agents is complex and multifactorial, but it is believed that due to the diuretic-like function, SGLT-2 inhibitors reduce glycemic-related toxicity, promote ketogenesis, and exert antihypertrophic, antifibrotic, and anti-remodeling properties. The benefits of dapagliflozin on cardiovascular death and worsening HF in patients with mildly reduced or preserved EF appeared especially pronounced in those with a greater degree of symptomatic impairment at baseline. Sotagliflozin led to a rise in the count of days patients were alive and not hospitalized (DAOH), which offers an extra patient-centered measure to assess the impact of the disease burden. The data in our article will help future researchers conduct large-scale trials with sotagliflozin to identify and implement it in the treatment of patients with HF as a mortality-reducing drug and to improve the quality of life for patients with HF.

## Introduction and background

On May 26, 2023, there was a remarkable update in the field of cardiology. On the basis of the phase three results of the effect of Sotagliflozin on Cardiovascular Events in Patients With Type 2 Diabetes Post Worsening Heart Failure (SOLOIST-WHF) and Sotagliflozin in Patients with Chronic Kidney Disease and Type 2 Diabetes (SCORED) trials, the U.S. Food and Drug Administration (FDA) approved sotagliflozin, making it the first dual sodium-glucose co-transporter 1 and 2 (SGLT 1 and 2) inhibitor authorized for the treatment of heart failure (HF) in both heart failure with preserved ejection fraction (HFpEF) and heart failure with reduced ejection fraction (HFrEF) [1]. Sotagliflozin has been approved to reduce cardiovascular mortality, hospitalization from HF, and urgent visits from HF in adults with heart failure, type 2 diabetes mellitus, chronic kidney disease, and other cardiovascular risk factors.

HF which is a leading cause of morbidity and mortality is a progressive and complex syndrome of left ventricular dysfunction. The term left ventricular ejection fraction (LVEF) categorizes systolic dysfunction, with LVEF >50% as normal [2].

Few advancements in the field of cardiometabolic medicine have had as significant an impact as sodium-glucose cotransporter-2 (SGLT-2) inhibitors [3]. SGLT-2 inhibitors (SGLT-2i) also called gliflozins differ in their relative inhibition of SGLT 1 and 2 receptors [4]. SGLT-2 inhibitors which were initially created as medications for lowering blood sugar levels showed significant outcomes such as reduced HF hospitalization and adverse kidney events in cardiovascular outcome trials [3].

SGLT-2 and SGLT-1 receptors, which are in charge of about 90% and 10%, respectively, of glucose reabsorption in the kidney proximal tubule, are inhibited by SGLTi to enhance urine glucose excretion. While SGLT-1 receptors are more extensively expressed and are primarily found in the colon and heart/skeletal muscle, SGLT-2 receptors are virtually entirely localized in the kidneys.

As of June 2023, 13 large-scale trials involving >90,000 participants, including the Empagliflozin Cardiovascular Outcome Event Trial in Type 2 Diabetes Mellitus Patients (EMPA-REG OUTCOME) and Dapagliflozin and Prevention of Adverse Outcomes in Heart Failure (DAPA-HF), explored SGLT-2 inhibitors in type-2 diabetes, HF, and kidney disease. While 12 trials reduced hospitalizations for HF, only these two demonstrated a significant reduction in cardiovascular mortality, highlighting uncertainties due to limited subgroup analysis in individual trials [3].

Sotagliflozin is a novel and unique diabetic medication that has dual inhibition of SGLT-1 and SGLT-2 receptors. SGLT-1 inhibition has been shown to delay postprandial intestinal glucose absorption and can also enhance levels of glucagon-like peptide-1 (GLP-1) and gastric inhibitory peptide (GIP). The ability to inhibit SGLT-1 molecules makes sotagliflozin different from the other SGLT-2 inhibitors which have been known for a while. The Emperor-reduced trial and DAPA-HF trials mentioned above have shown evidence of the cardiovascular benefits of SGLT-2 inhibitors. Both trials included patients with HFrEF, irrespective of their type-2 diabetes mellitus (T2DM) status. These trials demonstrated the decreased risk of hospitalization for HF and death from cardiovascular causes among patients with stable HF. However, there was no trial or data available on the effects of these drugs if initiated immediately after an episode of decompensated HF. The SOLOIST-WHF trial demonstrated the mortality benefits and significant cardiovascular outcomes of sotagliflozin when initiated immediately after a decompensation event. The results of this trial cannot be overlooked due to the large size of the combined trials, its randomized design, and the high standards with which it was conducted [5].

In light of the recent data from the SOLOIST-WHF and SCORED trials followed by the FDA approval of sotagliflozin, we conducted a systematic review to compare the cardiovascular mortality rates between sotagliflozin and dapagliflozin. Our primary outcome is to compare the differences in cardiovascular mortality or death rates in patients treated with sotagliflozin and dapagliflozin. The secondary outcomes include differences in HF hospitalization and the requirement for urgent admission for HF.

## Review

Methods

This systematic review adheres to the outlines prescribed by the Preferred Reporting Items for Systematic Review and Meta-Analysis (PRISMA) [6].

Search Strategy

To find relevant articles for our study, we conducted thorough searches using major research literature databases and search engines including PubMed, MEDLINE, PubMed Central, Google Scholar, Embase, and Cochrane Library. We used appropriate keywords and Medical Subject Headings (MeSH) to ensure comprehensive coverage and find articles related to our specific topic. To find the most pertinent articles, we combined the keywords in various combinations using Boolean operators "AND", "OR", and "NOT". This extensive approach helped us identify relevant and up-to-date literature for our study.

An overview of the databases that were reviewed for article collection and the corresponding search strategies employed are elaborated in Table 1.

**Table 1:** Database used for searching articles (along with search strategies and appropriate filters)

Type of Database	Keywords	Search Strategy	Filters Used	No. of records
PubMed Advanced Search	Sotagliflozin, Inpefa, Dapagliflozin, Farxiga, Heart Failure, Congestive Heart Failure, Mortality	Dapagliflozin OR Farxiga OR SGLT-2 inhibitors OR "Sodium-Glucose Transporter 2 Inhibitors" [Majr] OR "dapagliflozin" [Supplementary Concept] AND Sotagliflozin OR Inpefa OR Dual SGLT 1/2 inhibitors OR "(2S,3R,4R,5S,6R)-2-(4-chloro-3-(4-ethoxybenzyl) phenyl)-6-(methylthio) tetrahydro-2H-pyran-3,4,5-triol" [Supplementary Concept] AND Heart failure or Congestive Heart Failure OR (“Heart Failure/mortality"[Major] OR “Heart Failure/prevention and control"[Major])	Free full text,books and documents , Clinical Trial, Meta- Analysis, Randomized Controlled Trial, Review, Systematic Review, in the last 10 years, Humans	38
Google Scholar	SGLT-2 inhibitors, Dapagliflozin, Sotagliflozin, Heart Failure, Mortality	All in title: Sotagliflozin AND Heart Failure AND Mortality, All in title: Dapagliflozin AND Heart Failure	Published in the last 10 years	19
Embase	Sotagliflozin, Inpefa, Dapagliflozin, Farxiga, Heart Failure, Mortality	Sotagliflozin AND Dapagliflozin AND Heart Failure AND Mortality	Published in the last 10 years	167
Cochrane	Sotagliflozin, Dapagliflozin, Heart Failure, Mortality	Sotagliflozin AND Dapagliflozin AND Heart Failure AND Mortality	Only randomized controlled trials published in the last 10 years	3

Inclusion and Exclusion Criteria

The inclusion criteria for our review encompassed randomized controlled trials, systematic reviews, and meta-analyses that were published in the English language within the past 10 years, with a specific focus on the adult population comprising individuals aged 18 years and above and relevant to our research question. We excluded articles focusing on the pediatric population (<18 yrs.), case reports, letters to editors, animal studies, and unpublished or grey literature.

Analysis of Study Quality/Bias

After conducting a thorough assessment using standardized quality evaluation tools, we identified 11 out of the 12 selected studies as medium or high quality. These 11 studies were deemed suitable for inclusion in our review due to their credibility and robustness. Two different tools were employed for the assessment: the Assessment of Multiple Systematic Reviews (AMSTAR) tool for systematic reviews and meta-analyses, and the Cochrane risk-of-bias assessment tool for randomized controlled trials (RCTs).

A comprehensive evaluation of the quality of each study that was included in our review is shown in Table 2.

**Table 2:** Thorough assessment of the quality of each study included in our review AMSTAR 2: Assessment of Multiple Systematic Reviews 2 AMSTAR 2 checklist accepted score (>=70%): Minimum score 12 out of 16

Author	Publication Year	Report Type	Quality Assessment Tool	Score
Bhatt et al. [1]	2021	Randomized Controlled Trial	Cochrane Risk of Bias Assessment Tool	Low Risk of Bias
Perez et al. [4]	2023	Randomized Controlled Trial	Cochrane Risk of Bias Assessment Tool	Low Risk of Bias
Hasan et al. [7]	2023	Meta-Analysis	Assessment of Multiple Systematic Reviews 2 (AMSTAR 2)	15/16-High Quality
Ismayl et al. [8]	2023	Systematic Review and Meta-Analysis	AMSTAR 2	14/16-High Quality
Lee et al. [9]	2022	Systematic Review and Meta-Analysis	AMSTAR 2	15/16-High Quality
Cardoso et al. [10]	2021	Systematic Review and Meta-Analysis	AMSTAR 2	13/16-High Quality
Lu et al. [11]	2021	Meta-Analysis	AMSTAR 2	15/16-High Quality
Kosiborod et al. [12]	2023	Randomized Controlled Trial	Cochrane Risk of Bias Assessment Tool	Low Risk of Bias
Peikert et al. [13]	2022	Randomized Controlled Trial	Cochrane Risk of Bias Assessment Tool	Low Risk of Bias
Szarek et al. [14]	2021	Randomized Controlled Trial	Cochrane Risk of Bias Assessment Tool	Low Risk of Bias
McMurray et al. [15]	2019	Randomized Controlled Trial	Cochrane Risk of Bias Assessment Tool	Low Risk of Bias

Table 3 elaborates on the Cochrane risk of bias tool used to assess the quality of the RCTs we included.

**Table 3:** Comprehensive explanation of how we employed the Cochrane risk of bias tool to assess the quality of the Randomised Controlled Trials included in our study. Cochrane Risk of Bias Tool (Modified) For Quality Assessment of Randomised Controlled Trials “Yes” in all Domains would place a study at a “Low Risk of Bias” “No” in any of the domains would place a study at a “High Risk of Bias” “Unclear” in any of the domains would place the study at an “Unclear Risk of Bias”

Author	Bhatt et al. 2021 [1].	Perez et al. 2023 [4].	Kosiborod et al. 2023 [12].	Piekert et al. 2022 [13].	Szarek et al. 2021 [14].	McMurray et al. 2019 [15].
Random Sequence Generation: Was the allocation sequence already generated?	Yes	Yes	Yes	Yes	Yes	Yes
Allocation Concealment: Was the sequence generation adequately concealed before group assignments?	Yes	Yes	Yes	Yes	Yes	Yes
Blinding of participants: Was knowledge of the allocated interventions adequately hidden from the participants and personnel after participants were assigned to respective groups?	Yes	Yes	Yes	Yes	Yes	Yes
Blinding of Outcome Assessments: Was knowledge of the allocated interventions adequately hidden from the outcome assessors after participants were assigned to respective groups?	Yes	Yes	Yes	Yes	Yes	Yes
Incomplete Outcome Data: Were incomplete outcome data adequately addressed?	Yes	Yes	Yes	Yes	Yes	Yes
Selective reporting: Are reports of the study free of suggestion of selective outcome reporting	Yes	Yes	Yes	Yes	Yes	Yes
Other sources of bias: Was the study apparently free of other problems that could put it at risk of bias?	Yes	Yes	Yes	Yes	Yes	Yes
Risk of Bias	Low	Low	Low	Low	Low	Low

Results

In our initial search of PubMed, MEDLINE, PubMed Central, Embase, and Cochrane Library databases, we found a total of 227 articles. After applying relevant filters according to our eligibility criteria (last 10 years, human studies, English papers), we removed 15 duplicate articles, leaving us with 212 unique articles. These remaining articles were then screened by two independent investigators based on titles, abstracts, full-text, and detailed inclusion and exclusion criteria.

**Figure 1:**
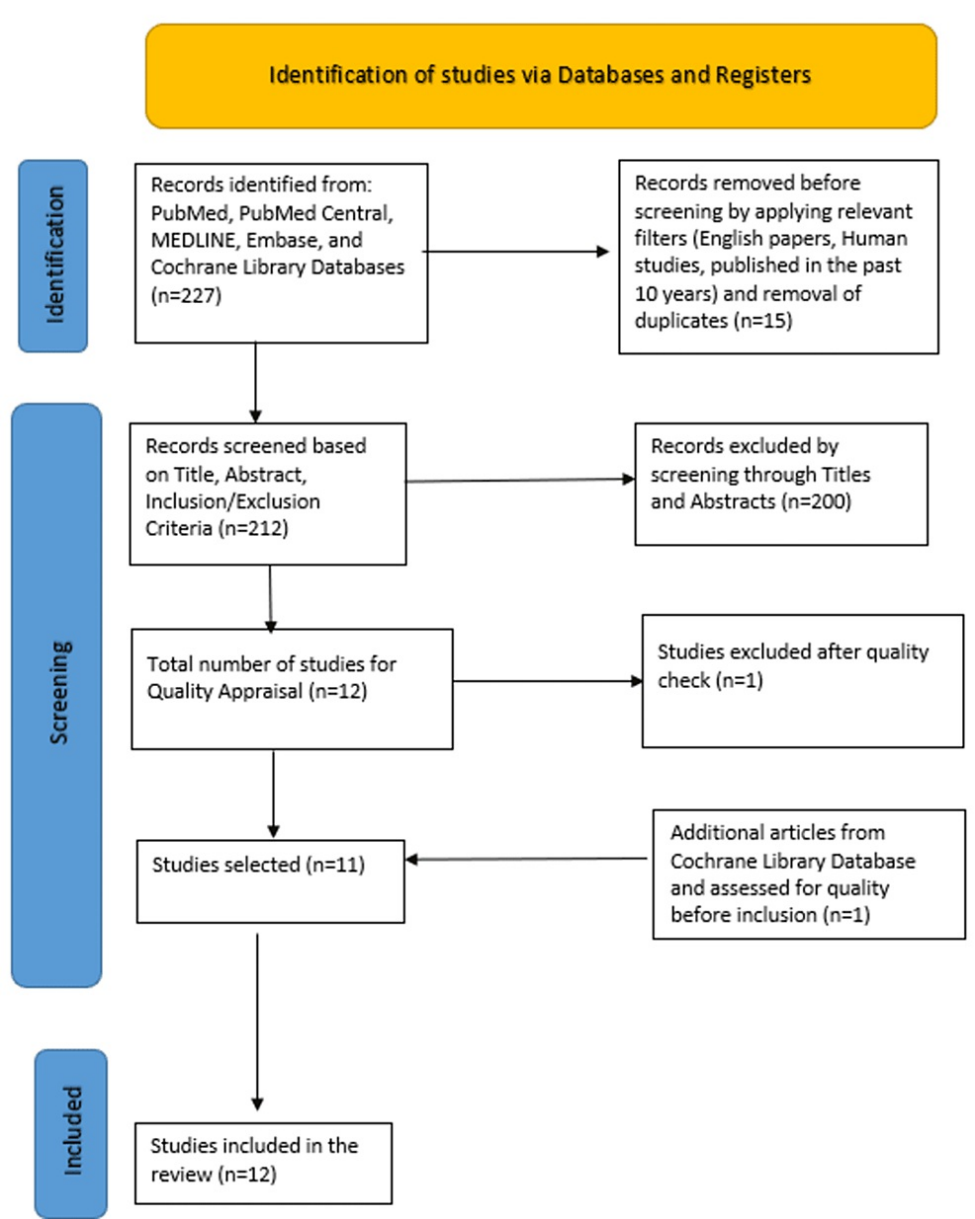
The screening process and quality assessment of the articles were documented using a PRISMA chart. PRISMA: Preferred Reporting Items for Systematic Reviews and Meta-Analyses

After this meticulous screening process, we were left with 11 articles. A single additional article was added by searching the relevant keywords on the Cochrane Library database. A total of 12 articles were chosen to undergo a comprehensive quality and bias assessment utilizing standardized quality assessment tools. Following the quality appraisal process, one study was excluded, and the final systematic review included a total of 11 studies. One study was excluded after quality appraisal, and the final 11 studies were included in this systematic review. Of the 11 included studies, three were systematic reviews and meta-analyses, two were purely meta-analyses and the remaining six articles were randomized controlled trials. The quality of the systematic review and meta-analyses were evaluated using the AMSTAR-2 checklist. The Cochrane Risk of Bias Assessment Tool was used for assessing the bias risk in the randomized controlled trials. The PRISMA 2020 flow diagram is depicted in Figure 1 [6].

Discussion

**Figure 2:**
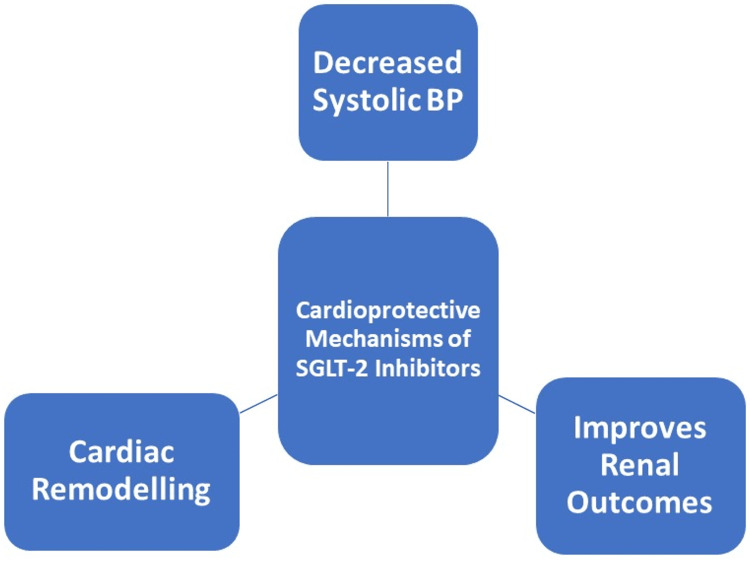
Cardioprotective effects of SGLT-2 inhibitors This is an original figure created by the first author NI. BP: Blood pressure; SGLT-2: sodium glucose co-transporter-2

HF is a significant global health concern, characterized by substantial morbidity and mortality [16]. SGLT-2 is a transport protein located in the kidney responsible for reabsorbing glucose from the glomerular filtrate back into the bloodstream. Some major cardiovascular outcome trials have shown the benefit of SGLT-2 inhibitors over placebo in major cardiovascular endpoints of mortality and hospitalizations for HF [10]. The inhibition of SGLT-2 causes glycosuria and natriuresis which contribute to decreased glucose levels (antidiabetic effect), blood pressure, and body weight. The pathophysiology behind the cardio-protection offered by these agents is complex and multifactorial but it is believed that due to the diuretic-like function, SGLT-2 inhibitors reduce glycemic-related toxicity, promote ketogenesis, and exert anti-hypertrophic, antifibrotic, and anti-remodeling properties shown in Figure 2 [16].

Several major cardiovascular outcome trials evaluated and demonstrated the beneficial cardioprotective effects of SGLT-2 inhibitors. The major ones were the Empagliflozin Cardiovascular Outcome Trial (EMPA-REG) trial which studied Empagliflozin, Canagliflozin Cardiovascular Assessment Study (CANVAS) which showed a significant reduction in HF hospitalization with Canagliflozin, the Dapagliflozin Effect on Cardiovascular Events Thrombolysis in Myocardial Infarction 58 (DECLARE-TIMI 58) trial and the most recent one being the effect of Sotagliflozin on Cardiovascular and Renal Events in Patients with Type 2 Diabetes and Moderate Renal Impairment Who Are at Cardiovascular Risk (SCORED) trial which demonstrated a significant reduction in the risk of death from cardiovascular causes or hospitalization for heart failure with the use of sotagliflozin [17].

Dual SGLT 1 and 2 Inhibition: Mechanisms

**Figure 3:**
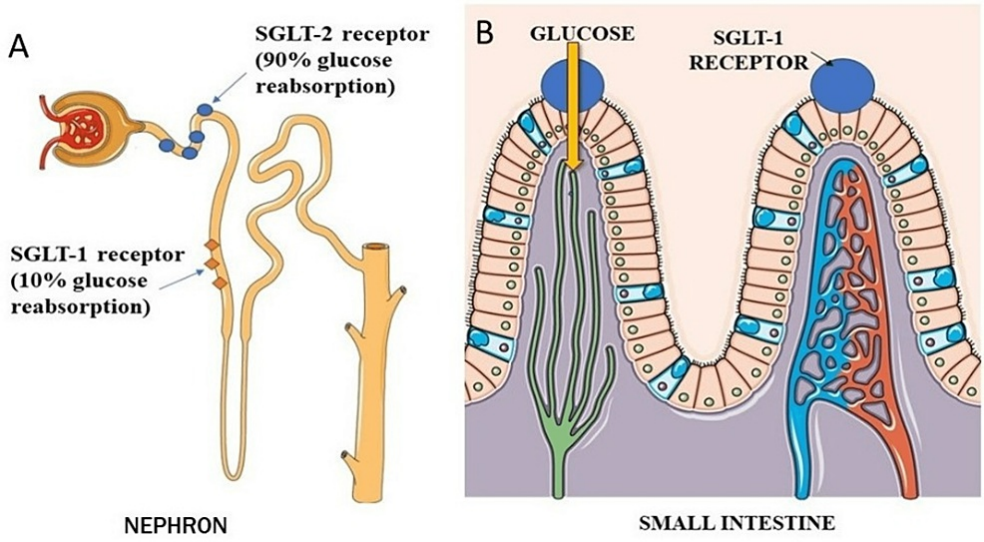
Sodium-glucose co-transporter (SGLT) 1 and 2 receptors in the nephron and small intestine Parts of the figure were drawn using pictures from Servier Medical Art. Servier Medical Art by Servier is licensed under a Creative Commons Attribution 3.0 Unported License (https://creativecommons.org/licenses/by/3.0/).

Sodium-glucose co-transporters can be classified based on their modulatory sites with SGLT-2 predominating in the kidney and SGLT-1 in the intestine. SGLT-1 transporters have dual functions in the body. They are involved in absorbing glucose from the small intestine during digestion, and they also reabsorb approximately 10% of the filtered glucose from the kidney’s proximal tubule back into the bloodstream [18]. The positioning of SGLT 1 and 2 receptors in the nephron and the small intestine is illustrated in Figures 3A, 3B.

The usual renal threshold for reabsorption of glucose is a serum glucose concentration of 180mg/dl. However, interestingly, in diabetics, the SGLT-2 protein becomes upregulated which may contribute to the worsening of the hyperglycemia. The inhibition of the SGLT-2 receptors prevents glucose reabsorption in the kidneys, thus allowing for better control of glucose in the diabetic population. Sotagliflozin is particularly unique for its dual ability to inhibit SGLT-1 and SGLT-2 receptors. SGLT-1 inhibition has been shown to cause a delay in postprandial intestinal absorption of glucose and increase levels of GLP-1 and GIP. This dual function along with the many beneficial cardiovascular outcomes discussed further make Sotagliflozin a novel drug of choice for patients with heart failure.

In an efficient and elaborate study done by Lee et al, comprising 52,607 patients from 10 trials, it was shown that in patients with heart failure, the SGLT-2 inhibitors with low selectivity towards SGLT-1 and SGLT-2 had a greater impact on reducing the risk of hospitalization for heart failure (HHF) compared to agents with higher selectivity [5,9].

Effect of SGLT-2 Inhibitors on Cardiovascular Outcomes in HF

According to the 2022 guidelines of the American Heart Association, American College of Cardiology, and Heart Failure Society of America, SGLT-2 inhibitors have been designated as Class I, level A drugs for the management of heart failure with reduced ejection fraction and as Class II A, level B for treating heart failure with mildly reduced ejection fraction (HFmEF; EF-41-49%) or preserved EF (HFpEF; LVEF-50%) [8]. While the main mechanism of action of drugs treating HFrEF acts on attenuating the overactivation of endogenous neurohormonal signals, very few options exist to treat HFmEF or HFpEF.

While earlier trials such as the DECLARE-TIMI 58 trial involving Dapagliflozin and Cardiovascular Outcomes Following Ertugliflozin Treatment in Type 2 Diabetes Mellitus Participants With Vascular Disease (VERTIS-CV) trial testing Ertugliflozin failed to demonstrate significant cardiovascular outcomes in patients with HF with ejection fraction >45%, we noticed that recent trials such as SOLOIST-WHF have shown significant outcomes in patients with HF and LVEF >50% with Sotagliflozin. However, the limited number of occurrences or events makes it unreliable to fully assess the treatment’s potential benefits [19].

Larger trials such as EMPEROR-PRESERVED (5988 patients) and DELIVER (6263 patients) are the two largest trials to date evaluating the benefits of SGLT-2 inhibitors in patients with HF with LVEF >40% [20,21]. Each of these trials individually showed a notable decrease in cardiovascular mortality and hospitalization for heart failure when using an SGLT-2 inhibitor, regardless of whether the patients had diabetes or not. A pooled analysis of six randomized controlled trials conducted by Ismayl et al. presumed that the significant reduction in HFH can be attributed to the effect of SGLT-2 inhibitors on the sympathetic nervous system, oxidative stress, reduced epicardial fat, the renin-angiotensin-aldosterone system which ultimately leads to improvement in contractility and distensibility of the heart [8,22,23].

Hasan et al. analyzed a total of 13 randomized controlled clinical trials including 75,287 patients demonstrating that SGLT-2 inhibitors significantly reduce the risk of hospitalization in patients with HF irrespective of their diabetes status but showed a significant reduction in mortality risk only in diabetics (Relative Risk (RR) =0.87,95% Confidence Interval (CI): 0.77-0.99) [7]. They attributed this occurrence in diabetics to the increased electrolyte free-water clearance and plasma osmolality which help in the removal of the extra interstitial fluid thus decreasing the circulatory overload on the failing heart. In non-diabetics, the non-significant reduction can be explained by the lack of deteriorating amount of glucose present in the circulation which contributes to the volume overload.

In an extensive meta-analysis conducted by Lu et al., which included eight large trials involving 16,640 patients with HF, SGLT-2 inhibitors were found to significantly decrease the risk of cardiovascular disease and hospitalization for heart failure (CVD/HHF) by 23% across a diverse range of heart failure patients [11]. In contrast to the previous study, subgroup analysis in this study demonstrated that SGLT-2i were effective in reducing cardiovascular outcomes in HF patients irrespective of their diabetic status. However, the trends were shown to be more strongly effective in HFpEF patients. This study had limitations of only including eight trials, thus still representing only a small sample size for pooling data. Secondly, they included only two studies in patients without diabetes and two in patients with HFpEF thus pointing out the need for caution while viewing the results of the subgroup analysis. A statistically significant 32% reduction in HHF (Hazard ratio (HR)-0.68,95% Confidence Interval (CI): 0.62-0.75) and a 15% reduction in cardiovascular death (HR-0.89,95% CI:0.76-0.94) was demonstrated with SGLT-2 inhibitors compared to placebo.

In a meta-analysis of 15 trials including over 20,000 patients conducted by Cardoso et al., SGLT-2 inhibitors were shown to significantly reduce all-cause mortality and cardiovascular mortality in patients with HF stratified by age, sex, race, renal function, diabetic status, the functional class of HF and ejection fraction [10]. The study however had certain limitations, one of them being that the pooled outcomes of SGLT-2 inhibitors under the same intervention group caused an inability to assess the differences in HF outcomes between different drugs particularly given the dual inhibition of SGLT-2 and SGLT-1 by Sotagliflozin which could not be assessed.

Table 4 provides a brief summary of the studies that compare SGLT-2 inhibitors with placebo in terms of cardiovascular mortality and HF hospitalization outcomes.

**Table 4:** A concise overview of the studies comparing SGLT-2 inhibitors with placebo in terms of cardiovascular mortality and heart failure hospitalization outcomes

Author and Year of Publication	Intervention studied	Number of patients included	Type of Study	Cardiovascular Mortality	Heart Failure Hospitalization (HFH)	Conclusion
Hasan et al., 2023 [7].	Sodium Glucose Co-transporter-2 inhibitors (SGLT-2i) vs Placebo	75,287 patients with Heart Failure (HF) with and without diabetes	Meta-Analysis	In patients with diabetes: Relative risk (RR)- 0.68, (95% Confidence IntervaI (CI): 0.63 to 0.74) and without diabetes (RR = 0.75, 95% CI: 0.62 to 0.89).	In patients with diabetes: Relative risk (RR)- 0.87 (95% Confidence Interval (CI) : 0.77-0.99) and without diabetes RR=0.93 (95% CI:0.70-1.23)	Sodium-Glucose Co-transporter-2 inhibitors (SGLT-2i) significantly reduce Heart Failure Hospitalization (HFH) irrespective of Diabetes Mellitus (DM) status but statistically significant reduction in cardiovascular mortality was only seen in diabetics.
Ismayl et al., 2023 [8].	SGLT-2 inhibitors vs Placebo	15,989 patients with Heart Failure (HF) and Left ventricular ejection fraction (LVEF) >40%	Systematic Review and Meta-Analysis of Randomized Controlled Trials	Cardiovascular Mortality: Hazard ratio (HR)- 0.74 (95% CI: 0.67-0.83)	Heart Failure Hospitalization: HR- 0.80; (95% CI: 0.74-0.86)	SGLT2i significantly reduce the combined risk of HFH or cardiovascular mortality
Lee et al., 2022 [9].	SGLT-2 inhibitors with high SGLT 1 / 2 selectivity and low selectivity vs Placebo	52,607 patients	Systematic Review and Meta-Analysis of Randomized Trials	RR- 0.72 (95% CI: 0.66–0.79) in both HF and non-HF groups. Only high-selectivity inhibitors have an effect in patients without HF.	RR- 0.91 (95% CI: 0.83–0.99) in patients with HF, but insignificant in those without HF.	In heart failure patients, SGLT2 inhibitors with low SGLT2/SGLT1 selectivity were more effective in reducing the risk of HHF compared to those with high selectivity, while this effect was not seen in non-heart failure patients.
Cardoso et al., 2021 [10].	SGLT-2 inhibitors vs Placebo	20,241 patients	Systematic review and meta-analysis	HR- 0.69 (95% CI: 0.62-0.76)	HR-0.86 (95% CI: 0.78-0.96)	SGLT-2 inhibitors reduce the composite of HFH, and CVD across multiple subgroups irrespective of age, gender, race, eGFR, and EF status.
Lu et al., 2021 [11].	SGLT-2 inhibitors vs Placebo	16,460 patients with heart failure	Meta-analysis of randomized controlled trials	HR- 0.68, (95% CI: 0.62–0.75)	HR- 0.85, (95% CI: 0.76–0.94)	SGLT-2i were robustly effective in reducing cardiovascular outcomes in Heart failure with reduced ejection fraction (HFrEF) patients as well as HF patients with or without Type 2 Diabetes Mellitus (T2DM), and displayed a strong trend to be effective in Heart Failure with preserved ejection fraction (HFpEF).

Cardiovascular Mortality and Other Significant Outcomes With Sotagliflozin 

Bhatt et al. conducted the effect of sotagliflozin on Cardiovascular Events in Patients with Type 2 Diabetes Post-Worsening Heart Failure (SOLOIST-WHF) trial, a multicenter, double-blinded, randomized, and placebo-controlled study. This trial aimed to compare the effects of sotagliflozin, a dual SGLT1 and SGLT2 inhibitor, with placebo in patients with type 2 diabetes and reduced or preserved left ventricular ejection fraction who had recently been hospitalized due to worsening HF [1,14]. During the study, 1222 patients were randomly assigned, with 608 in the sotagliflozin group and 614 in the placebo group. The patients were then monitored for a period of nine months.

The study population included patients with type 2 diabetes who were recently hospitalized for worsening of HF. The main focus or primary endpoint of the study was to measure the combined total of cardiovascular deaths and hospitalizations or urgent visits related to heart failure. In the sotagliflozin group, the primary endpoint event rate was 51.0 per 100 patient-years, while in the placebo group, it was 76.3 per 100 patient-years (HR-0.67;95% CI: 0.52-0.85; P<0.001). When the total number of deaths that occurred due to cardiovascular causes and hospitalizations for HF was studied as a secondary endpoint excluding the urgent visits for heart failure, the results obtained were statistically significant and similar to the results of the primary endpoint (HR-0.68;95% CI: 0.53-0.88) [1].

A summary of the randomized controlled trials assessing sotagliflozin is presented in Table 5.

**Table 5:** Summary of the randomised controlled trials studying sotagliflozin SOTA-P-CARDIA: Sotagliflozin in Heart Failure With Preserved Ejection Fraction Patients; SOLOIST-WHF: Effect of Sotagliflozin on Cardiovascular Events in Patients with Type 2 Diabetes Post-Worsening Heart Failure Trial; HFpEF: Heart Failure with Preserved Ejection Fraction; DAOH: Days alive and out of hospital

Author, Year of Publication and, name of the trial	Intervention Studied	Number of patients included	Name of the randomized controlled trial (RCT)	Primary end-point being studied	Results	Conclusion
Bhatt et al., 2021 [1].	Sotagliflozin Vs Placebo	1222 patients	Effect of sotagliflozin on Cardiovascular Events in Patients with Type 2 Diabetes Post-Worsening Heart Failure (SOLOIST-WHF) Trial	The main objective or primary endpoint was to measure the total number of deaths resulting from cardiovascular causes and hospitalizations and urgent visits for heart failure including both first-time occurrences and subsequent events.	The rate (the number of events per 100 patient-years) of primary end-point events was lower in the sotagliflozin group than in the placebo group (51.0 vs. 76.3; Hazard ratio (HR)- 0.67; 95% confidence interval [CI]: 0.52 to 0.85; P<0.001).	Patients with diabetes who received sotagliflozin therapy either before or shortly after discharge, experienced a notably reduced total number of deaths from cardiovascular causes and hospitalization as well as urgent visits for heart failure, compared to those who received placebo.
Perez et al., 2023 [4].	Sotagliflozin Vs Placebo	82 non-diabetic patients with HFpEF (>50%)	Sotagliflozin in Heart Failure With Preserved Ejection Fraction Patients (SOTA-P-CARDIA) Trial	The study examined changes in the left ventricular mass by cardiac magnetic resonance from the time of randomization to the end of the study.	The trial is still ongoing and the expected benefits are that the demonstrated benefits of sotagliflozin in diabetic patients will also be demonstrated in non-diabetic HFpEF patients.	Novelties of this trial: a) use of dual SGLT ½ inhibitor b) first trial to include non-diabetic HFpEF patients c)random assessment of EF using MRI.
Szarek et al., 2021 [14].	Sotagliflozin Vs Placebo	1222 patients with type 2 diabetes and reduced or preserved ejection fraction who were recently hospitalized for worsening heart failure	Effect of sotagliflozin on Cardiovascular Events in Patients with Type 2 Diabetes Post-Worsening Heart Failure (SOLOIST-WHF) Trial	Prespecified Poisson regression models were employed to analyze the metrics of "days alive and out of the hospital" (DAOH) and its converse, "days dead and days in the hospital".	The rate of "days alive and out of the hospital" (DAOH) in the sotagliflozin group was 3% higher than in the placebo group (rate ratio [RR]- 1.03 [95% CI: 1.00 to 1.06]; P = 0.027.	Sotagliflozin treatment led to an increase in "days alive and out-of-hospital" (DAOH), which is a patient-centered outcome that helps capture the overall burden of the disease.

The SOLOIST-WHF trial established that in patients with diabetes and worsening heart failure, the administration of Sotagliflozin, a dual SGLT 1 and 2 inhibitor, was associated with a notably reduced total number of cardiovascular deaths when compared to the placebo group. There was also a statistically significant reduction in hospitalizations and urgent visits for heart failure.

Following the positive findings of the previous study, Szarek et al. conducted a study to assess whether the use of sotagliflozin improved the prespecified efficacy outcome of "days alive and out of the hospital" (DAOH) in the SOLOIST-WHF trial [14]. They discovered that fewer patients in the Sotagliflozin group experienced multiple hospitalizations compared to the placebo group (16.3 % vs. 22.1 %). In the sotagliflozin group, there were 64 deaths, while in the placebo group, there were 76 deaths. The rate of "days alive and out of the hospital" (DAOH) was 3% higher in the sotagliflozin group compared to the placebo group (rate ratio (RR):1.03, (95% CI: 1.00 to 1.06); P=0.027). The main reason for this difference was a decrease in the rate of mortality (days dead) rather than a decrease in the rate of hospitalization for any cause (days hospitalized). The sotagliflozin group had 2.9 days longer “days alive and out of the hospital” than the placebo group.

Another ongoing major trial studying sotagliflozin is the Sotagliflozin in Heart Failure With Preserved Ejection Fraction Patients (SOTA-P-CARDIA) trial which was accepted in May 2023. The study aims to demonstrate the benefits of sotagliflozin in non-diabetic HFpEF patients when administered on top of their prescribed HF treatment. The primary endpoint that will be assessed would be the reduction in the left ventricular mass by Sotagliflozin assessed by cardiac MRI at baseline vs. six months and compared with placebo. The study is ongoing and the expected results are that the previous results and benefits of sotagliflozin in diabetic patients would also be seen in non-diabetic HFpEF patients.

Some of the unique features of the SOLOIST-WHF trial are the inclusion of hospitalized patients as well as patients with preserved ejection fractions or LVEF>50% which was different from previous trials which only included patients with reduced ejection fractions. However, some significant limitations were the early termination and the small sample size of the subgroup which had LVEF>50%. In the comparatively larger Sotagliflozin in Patients with Diabetes and Chronic Kidney Disease (SCORED) trial, sotagliflozin resulted in a 26% lower risk of the same redefined outcome as SOLOIST-WHF [24]. Thus, there is abundant cardiovascular evidence to suggest that Sotagliflozin definitely has great potential to be added as an additional therapeutic tool in the management of patients with decompensated heart failure irrespective of their ejection fraction [5].

Thus, the SOLOIST-WHF and the SCORED trials were two large-scale randomized clinical trials that evaluated the cardiovascular effects of the dual SGLT 1 and 2 inhibitor Sotagliflozin and have demonstrated the cardiovascular benefits of mortality reduction with Sotagliflozin.

Comparative Mortality and Other Significant Outcomes With Dapagliflozin

One of the noteworthy trials investigating Dapagliflozin was the Dapagliflozin Evaluation to Improve the Lives of Patients With Preserved Ejection Fraction Heart Failure (DELIVER) trial conducted by Kosiborod et al. [12]. The unique feature of this trial was the usage of the Kansas City Cardiomyopathy Questionnaire (KCCQ) and using the scores obtained to stratify the patients in the trial. The KCCQ is a 23-item, validated, and self-administered questionnaire that helps to quantify HF-related symptoms such as frequency, severity, function, and quality of life. This randomized clinical trial involved a total of 5795 patients and aimed to study the effects of dapagliflozin on clinical outcomes. It was noted that the effect of Dapagliflozin in the reduction of cardiovascular death and worsening of heart failure was much more pronounced in patients who had a greater baseline symptom burden which was determined based on their KCCQ -Total Symptom Score (TSS). The primary outcome was to evaluate the composite of cardiovascular death or events of worsening of heart failure which could be an unplanned heart failure hospitalization or urgent heart failure visit requiring the use of intravenous medications. There was an extensive follow-up period with visits at every four months up to 16 months, and every four months after that. Of the 5795 patients who had KCCQ data available at baseline,5278 patients had their KCCQ evaluated at the one-month follow-up visit, 4808 had their KCCQ evaluated at four months and 4411 had theirs evaluated at the eighth-month visit. In the DELIVER trial, a significant observation was made: patients with lower baseline Kansas City Cardiomyopathy Questionnaire-Total Symptom Score (KCCQ-TSS) experienced higher rates of cardiovascular death or worsening of heart failure. The rates were 7.8, 5.6, and 4.8 per 100 patient-years in patients with KCCQ-TSS tertiles of <63, 63-84, and >84, respectively, with a p-value of <0.0001. The main finding of this study was that the significant reduction in cardiovascular death or worsening HF observed with Dapagliflozin was more pronounced in patients who had a greater burden of symptoms at baseline (lowest to highest KCCQ-TSS tertile: HR: 0.70 [95% CI: 0.58-0.84]; 0.81 [95% CI: 0.65-1.01]; 1.07 [95% CI: 0.83-1.37]; p interaction=0.026). A graded relationship was observed with a lower KCCQ-TSS score at baseline and a greater reduction in the primary endpoint of cardiovascular (CV) death or worsening HF with dapagliflozin as compared to placebo. The DELIVER trial was among the initial and most extensive global outcomes studies to reveal a decrease in symptom burden caused by heart failure in patients with mildly reduced or preserved ejection fraction. This trial demonstrated significant evidence of the positive effects of Dapagliflozin on managing heart failure symptoms in this group of patients.

As part of the DELIVER trial, Peikert et al. conducted another study focusing on the safety and efficacy outcomes in different age categories of patients with heart failure and mildly reduced or preserved ejection fraction [13]. This study randomized 6263 patients from ages 40 to 99 years across 350 sites in 20 countries. It was observed that crude event rates for the primary outcome of worsening heart failure events or cardiovascular death and its components did not significantly differ between the age categories, while there was an increase in the rate of all-cause mortality with age. Dapagliflozin was shown to reduce the risk of the primary outcomes mentioned above irrespective of age categories (P interaction=0.95). Similarly, the treatment effect of Dapagliflozin compared with placebo on cardiovascular death, worsening HF events (either a hospitalization for HF or urgent HF visit) and all-cause mortality did not significantly differ across the age categories (p interaction >0.7 for all). This was contrasting to previous trials such as DAPA-HF which observed a more prominent increase in risk with age. The baseline prevalence of atrial fibrillation and atrial flutter was higher in older patients, with lower body mass index, heart rate, diastolic blood pressure, and HbA1c levels observed with increasing age. As demonstrated in previous HF trials, the absolute baseline NT-pro BNP levels in older age groups correlate with the possibility of higher levels of myocardial stress in this population. Most older patients with HFpEF were found to have smaller left ventricular sizes, higher estimated ejection fractions, and distinct cardiac remodeling patterns when compared to their younger counterparts. This was also demonstrated in the DELIVER trial where older patients had the highest average LVEF and more patients had LVEF > 60 %. Thus, the data from the DELIVER trial showcase the fact that neither age nor ejection fraction attenuates the benefits of SGLT-2 inhibition.

The Dapagliflozin And Prevention of Adverse-outcomes in Heart Failure trial (DAPA-HF) is an international, multi-center, randomized double-blinded controlled trial to determine the safety and efficacy of SGLT-2 inhibitor dapagliflozin added to the conventional heart failure therapy in patients with heart failure with reduced ejection fraction [25]. The DAPA-HF trial demonstrated statistically significant benefits, including reductions in morbidity and mortality, when an SGLT-2 inhibitor was incorporated into a guideline-directed therapy for heart failure management. This was definitely marked as a dramatic evolution from being a glucose-lowering therapy to becoming an effective medication for heart failure. The primary endpoint of composite death from cardiovascular causes or worsening heart failure was significantly reduced by 26% (16.3 vs 21.2 %; HR-0.74; 95% CI: 0.65-0.85; p<0.001). In the DAPA-HF trial, there was a notable 30% reduction in worsening heart failure events among patients receiving the SGLT-2 inhibitor as part of their treatment (10.0 vs 13.7%; HR 0.70; 95% CI: 0.59-0.83; p<0.00004) and 18% reduction in cardiovascular mortality (9.6 vs 11.5%; HR: 0.82; 95% CI: 0.69-0.98; p=0.029). The secondary outcome of HFH or death from CV causes was found to be lower in the dapagliflozin group (16.1 vs 20.9%; HR 0.75; 95% CI: 0.65-0.85; p<0.001).

Thus, extensive research with SGLT-2 inhibitors has helped us arrive at this position with HF management.

A comprehensive summary of the randomized controlled trials assessing dapagliflozin is presented in Table 6.

**Table 6:** Summary of randomised controlled trials studying dapagliflozin DELIVER: Dapagliflozin Evaluation to Improve the Lives of Patients With Preserved Ejection Fraction Heart Failure; DAPA-HF: Dapagliflozin And Prevention of Adverse Outcomes in Heart Failure Trial; KCCQ: Kansas City Cardiomyopathy Questionnaire; TSS: Total Symptom Score; PLS: Physical Limitations Score; CSS: Clinical Summary Score; OSS: Overall Summary Score

Author and Year of Publication	Intervention being studied	Number of patients included	Name of Randomised Controlled Trial (RCT)	Results	Conclusion
Kosiborod et al., 2023 [12].	Dapagliflozin vs Placebo	5795 patients	Dapagliflozin Evaluation to Improve the Lives of Patients With Preserved Ejection Fraction Heart Failure (DELIVER)	Lowest-to-highest Kansas City Cardiomyopathy Questionnaire-Total Symptom Score (KCCQ-TSS) tertile: HR: 0.70 [95% CI: 0.58-0.84]; 0.81 [95% CI: 0.65-1.01]; 1.07 [95% CI: 0.83-1.37]; P interaction=0.026). At 8 months, Dapagliflozin was found to improve Kansas City Cardiomyopathy Questionnaire-Total Symptom Score (KCCQ-TSS), Physical Limitation Scale (PLS), Clinical Summary Score (CSS), and Overall Summary Score (OSS) (2.4, 1.9, 2.3, and 2.1 points higher vs placebo); P < 0.001 for all	Dapagliflozin showed stronger effects in reducing cardiovascular death and worsening heart failure in patients with higher baseline symptom burden
Peikert et al., 2022 [13].	Dapagliflozin vs Placebo	6263 patients	Dapagliflozin Evaluation to Improve the Lives of Patients With Preserved Ejection Fraction Heart Failure (DELIVER)	No significant differences in predefined safety outcomes between patients across all age categories.	Among patients with heart failure and mildly reduced or preserved ejection fraction, Dapagliflozin reduced the combined risk of cardiovascular death or worsening heart failure events across all age groups, including older patients aged 75 years and above
McMurray et al., 2019 [15].	Dapagliflozin vs Placebo	4744 patients	Dapagliflozin And Prevention of Adverse Outcomes in Heart Failure Trial (DAPA-HF)	The primary outcome, which includes cardiovascular death, hospitalization for heart failure, or urgent heart failure visit, occurred in 16.3% of the dapagliflozin group and in 21.2% of the placebo group (p < 0.001).	Dapagliflozin was linked to a decrease in cardiovascular deaths and heart failure events.

Limitations

This systematic review encounters some limitations. Firstly, the majority of the information provided in this review is based on articles, studies, and clinical trials published within the last 10 years. Furthermore, this review relied solely on free full-text articles accessible from various databases to gather information. Publications in languages other than English were excluded during the screening process for this review. Studies involving minors including the pediatric population (i.e. individuals below 18 years of age), were not included in this review. Studies in which animal models have been used to test the efficacy and safety of SGLT-2 inhibitors have been excluded from the review. Since some trials with sotagliflozin are still ongoing, more data are required to make definitive conclusions. In certain included clinical trials, subgroup analyses were conducted without adjusting for multiple comparisons. The prespecified inclusion and exclusion criteria in some trials may have limited the enrolment of some very high-risk patients which could affect the generalizability of the results. Lastly, we must account for information bias due to the non-availability of certain data collected from various databases worldwide.

## Conclusions

This systematic review was carried out to compare the cardiovascular mortality rates between sotagliflozin and dapagliflozin in patients with heart failure. With the significant FDA approval of the dual SGLT 1 and 2 inhibitor sotagliflozin, we conducted a review that compared the cardiovascular mortality rates between sotagliflozin and dapagliflozin. In our review, we found that sotagliflozin if initiated immediately after an episode of decompensation of HF significantly reduces the cardiovascular mortality rates. Dapagliflozin's impact on reducing cardiovascular death or worsening heart failure was more prominent in patients with a greater baseline symptom burden. We did not find any comparative review articles focusing on sotagliflozin and we hope that this article helps provide future researchers with the information they need to conduct more large-scale trials involving this unique medication. We also advocate conducting more clinical trials in the context of studying the benefits of sotagliflozin in patients with type 1 diabetes and low glomerular filtration rates to obtain more information on the safety and efficacy of the drug.

## References

[1] Bhatt DL, Szarek M, Steg PG (2021). Sotagliflozin in patients with diabetes and recent worsening heart failure. N Engl J Med.

[2] van Riet EE, Hoes AW, Wagenaar KP, Limburg A, Landman MA, Rutten FH (2016). Epidemiology of heart failure: the prevalence of heart failure and ventricular dysfunction in older adults over time. A systematic review. Eur J Heart Fail.

[3] Wiviott SD, Berg DD (2023). SGLT2 inhibitors reduce heart failure hospitalization and cardiovascular death: clarity and consistency. J Am Coll Cardiol.

[4] Pérez MS, Rodríguez-Capitán J, Requena-Ibáñez JA (2023). Rationale and design of the SOTA-P-CARDIA Trial (ATRU-V): Sotagliflozin in HFpEF patients without diabetes. Cardiovasc Drugs Ther.

[5] Shah SR, Ali A, Ikram S (2021). Sotagliflozin and decompensated heart failure: results of the SOLOIST-WHF trial. Expert Rev Clin Pharmacol.

[6] Page MJ, McKenzie JE, Bossuyt PM (2021). The PRISMA 2020 statement: an updated guideline for reporting systematic reviews. BMJ.

[7] Hasan MT, Awad AK, Shih M, Attia AN, Aboeldahab H, Bendary M, Bendary A (2023). Meta-analysis on the safety and efficacy of sodium glucose cotransporters 2 inhibitors in patients with heart failure with and without diabetes. Am J Cardiol.

[8] Ismayl M, Abbasi MA, Al-Abcha A, El-Am E, Lundgren S, Goldsweig AM, Anavekar NS (2023). Sodium-glucose cotransporter-2 inhibitors in heart failure with mildly reduced or preserved ejection fraction: a systematic review and meta-analysis of randomized controlled trials. Curr Probl Cardiol.

[9] Lee MC, Hua YM, Yang CT (2022). Clinical efficacy of SGLT2 inhibitors with different SGLT1/SGLT2 selectivity in cardiovascular outcomes among patients with and without heart failure: a systematic review and meta-analysis of randomized trials. Medicine (Baltimore).

[10] Cardoso R, Graffunder FP, Ternes CM, Fernandes A, Rocha AV, Fernandes G, Bhatt DL (2021). SGLT2 inhibitors decrease cardiovascular death and heart failure hospitalizations in patients with heart failure: a systematic review and meta-analysis. EClinicalMedicine.

[11] Lu Y, Li F, Fan Y, Yang Y, Chen M, Xi J (2021). Effect of SGLT-2 inhibitors on cardiovascular outcomes in heart failure patients: a meta-analysis of randomized controlled trials. Eur J Intern Med.

[12] Kosiborod M, Bhatt DL, Szarek M, Stet PG, Pitt B (2023). Effects of SGLT 1-2 inhibitor sotagliflozin on symptoms, physical limitations and quality of life in patients with worsening heart failure: results from the SOLOIST trial. J Am Coll Cardiol.

[13] Peikert A, Martinez FA, Vaduganathan M (2022). Efficacy and safety of dapagliflozin in heart failure with mildly reduced or preserved ejection fraction according to age: the DELIVER trial. Circ Heart Fail.

[14] Szarek M, Bhatt DL, Steg PG (2021). Effect of sotagliflozin on total hospitalizations in patients with type 2 diabetes and worsening heart failure: a randomized trial. Ann Intern Med.

[15] McMurray JJ, DeMets DL, Inzucchi SE (2019). A trial to evaluate the effect of the sodium-glucose co-transporter 2 inhibitor dapagliflozin on morbidity and mortality in patients with heart failure and reduced left ventricular ejection fraction (DAPA-HF). Eur J Heart Fail.

[16] McMurray JJV, Solomon SD, Inzucchi SE (2019). Dapagliflozin in patients with heart failure and reduced ejection fraction. N Engl J Med.

[17] Al Rifai M, Newby LK, Nair AP, Misra A, Rogers JG, Fedson S, Virani SS (2022). SGLT-2 inhibitors for patients with heart failure: what have we learned recently?. Curr Atheroscler Rep.

[18] Vallianou NG, Christodoulatos GS, Kounatidis D, Dalamaga M (2021). Sotagliflozin, a dual SGLT1 and SGLT2 inhibitor: In the heart of the problem. Metabol Open.

[19] Kato ET, Silverman MG, Mosenzon O (2019). Effect of dapagliflozin on heart failure and mortality in type 2 diabetes mellitus. Circulation.

[20] Solomon SD, McMurray JJ, Claggett B (2022). Dapagliflozin in heart failure with mildly reduced or preserved ejection fraction. N Engl J Med.

[21] Anker SD, Butler J, Filippatos G (2021). Empagliflozin in heart failure with a preserved ejection fraction. N Engl J Med.

[22] Hallow KM, Helmlinger G, Greasley PJ, McMurray JJ, Boulton DW (2018). Why do SGLT2 inhibitors reduce heart failure hospitalization? A differential volume regulation hypothesis. Diabetes Obes Metab.

[23] Anker SD, Khan MS, Shahid I, Filippatos G, Coats AJ, Butler J (2021). Sodium-glucose co-transporter 2 inhibitors in heart failure with preserved ejection fraction: reasons for optimism. Eur J Heart Fail.

[24] Volpe M, Patrono C (2021). The value of sotagliflozin in patients with diabetes and heart failure detracted by an unexpected ending. Eur Heart J.

[25] Kaplinsky E (2020). DAPA-HF trial: dapagliflozin evolves from a glucose-lowering agent to a therapy for heart failure. Drugs Context.

